# GLDC interacts with VPS34 to inhibit tumorigenesis and epithelial-mesenchymal transition in hepatocellular carcinoma

**DOI:** 10.1016/j.pscia.2025.100072

**Published:** 2025-04-25

**Authors:** Zan Song, Hao Dong, Kailing Zhang, Bingke Qiao, Leilei Li, Zhicheng Zhang, Zhili Fan, Jing Li, Yu Li, Mengfei Liu, Ying Liu, Xinyu Gu, Tao Zhang

**Affiliations:** a Institute of Immunopharmaceutical Sciences, State Key Laboratory of Discovery and Utilization of Functional Components in Traditional Chinese Medicine, NMPA Key Laboratory for Technology Research and Evaluation of Drug Products, School of Pharmaceutical Sciences, Cheeloo College of Medicine, Shandong University, Jinan, Shandong, China; b Department of Pharmacy, Tengzhou Central People's Hospital, Shandong, China

**Keywords:** GLDC, Acetylation, VPS34, Epithelial-mesenchymal transition, HCC

## Abstract

Hepatocellular carcinoma (HCC) accounts for 90% of primary liver cancer with high mortality and limited therapeutic strategy. Glycine decarboxylase (GLDC) is the key limiting enzyme in glycine breakdown metabolism and acts as oncogene or tumor suppressor to impact tumor onset and progression in a context dependent manner. However, the underlying mechanism of GLDC on autophagy and progression is largely unexplored in HCC. Here, we showed that GLDC overexpression inhibited cell proliferation, cell migration and promoted cell senescence and autophagy in HCC. Intriguingly, induced GLDC remarkably attenuated epithelial-mesenchymal transition (EMT) progress and tumor growth *in vitro* and *in vivo*. Mechanically, we demonstrated that GLDC upregulated VPS34 protein and enhanced its interaction with VPS34, thus promoting the association of VPS34 with Beclin1/ATG14 complex and autophagy induction in HCC. Importantly, GLDC acetylation at K514 promoted interaction of GLDC-VPS34, whereas GLDC acetylation-dead mutant K514R abolished their binding. Furthermore, GLDC protein was decreased in HCC tissues compared with para-tumor tissues and reduced GLDC was significantly correlated with poor prognosis of patients. In conclusion, we unveil the key regulatory role of GLDC in autophagy and HCC progression through VPS34 and provide a potential strategy for HCC therapy.

## Highlights

• GLDC induces cellular senescence and suppresses cell proliferation.
• The interaction between GLDC and VPS34 promotes autophagy.
• Acetylation of GLDC at K514 enhances its interaction with VPS34.

## Graphical abstract

**Figure:**
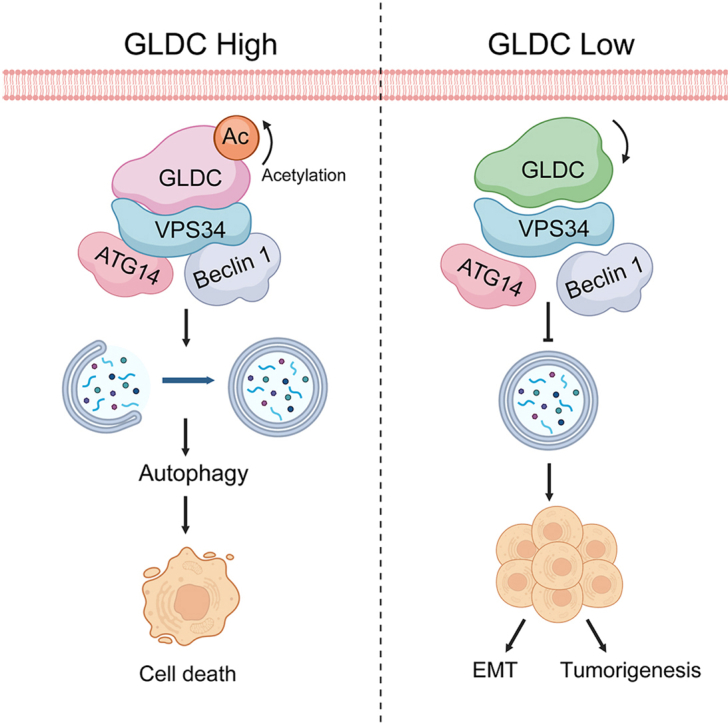

## Abbreviations:

GLDC: Glycine decarboxylase
VPS34: Vacuolar protein sorting 34
GCS: Glycine cleavage system
HCC: Hepatocellular carcinoma
IHC: Immunohistochemistry
co-IP: Co-immunoprecipitation
β-gal: β-galactosidase
TCGA: The Cancer Genome Atlas
NSCLC: non-small cell lung cancer
UVRAG: UV radiation resistance associated
ATG14: beclin-1-associated autophagy-related key regulator (Barkor)
KEGG: Kyoto Encyclopedia of Genes and Genomes
EMT: epithelial-mesenchymal transition
LC3B: microtubule associated protein 1 light chain 3 beta
EBSS: Earle's Balanced Salt Solution
Baf-A1: Bafilomycin A1

## 1 Introduction

According to Global Cancer Statistics 2022, the incidence rate of liver cancer ranks sixth and the mortality rate ranks third among malignant tumors [1]. Approximately 90% of primary liver cancers are hepatocellular carcinoma (HCC) [2]. In recent years, molecular targeted therapy has gradually become one of the effective means for clinical systemic treatment of HCC. However, only sorafenib and lenvatinib have been approved as first-line targeted drugs [3,4]. Due to the high heterogeneity of liver cancer, targeted drugs face several challenges, such as low response rates and drug resistance [5]. Therefore, it is imperative to elucidate the molecular mechanism of HCC and explore novel drug targets.

Glycine, a non-essential amino acid, is mainly involved in synthesis of protein and nucleotides [6,7]. Moreover, glycine links one-carbon metabolism, which plays a crucial role in tumor development, oxidative stress, metabolic rewiring, and immune regulation [[8], [9], [10], [11]]. Glycine breakdown primarily occurs through the glycine cleavage system (GCS) mediated by the core rate-limiting enzyme glycine decarboxylase (GLDC) [12]. However, the investigation on GLDC in tumor development is limited, and the role of GLDC is context-dependent in different cancer types. GLDC acts as a driver gene, which significantly promotes tumor initiation in non-small cell lung cancer (NSCLC) [13]. Similarly, aberrantly increased GLDC expression is associated with poor prognosis and accelerates cell proliferation, invasion, metastasis and immune escape in multiple cancers, including neuroblastoma, glioma and prostate cancer [[14], [15], [16]]. Conversely, GLDC exhibits reduced expression and inhibits cell migration and invasion in gastric carcinoma and HCC [[17], [18], [19]].

Autophagy is a multistep metabolic process, which maintains cell homeostasis through promoting the recycling of cellular components [20,21]. VPS34 (also known as phosphatidylinositol 3 kinase class III, PI3KC3) complexes play important roles in autophagy initiation and progression [22,23]. Autophagy specific VPS34 complex I is composed by VPS34, Beclin1, ATG14 and VPS15, which is involved in autophagosome formation and maturation [24,25]. In HCC, autophagy might serve as a tumor suppressor, enhances cell death and attenuates cell malignant characteristics, including proliferation, epithelial-mesenchymal transition (EMT) and metastasis [26,27]. However, the mechanism by which GLDC modulates autophagy and HCC progression remains unclear.

In this work, we demonstrated that GLDC overexpression induced cell senescence and inhibited cell proliferation, migration and tumor growth in HCC. GLDC interacted with VPS34 protein and further promoted protein-protein interactions of VPS34 autophagy complex to elicit autophagy. Notably, GLDC K514 acetylation enhanced the interaction between GLDC and VPS34. Furthermore, GLDC expression was down-regulated and correlated with poor prognosis in HCC.

## 2 Materials and methods

### 2.1 Cell lines and reagents

Huh7, Hep3B, HepG2, SK-Hep-1, Li7 and 293T cell lines were obtained from the Cell Bank in the Chinese Academy of Sciences. CLC13 cell line was obtained from Bio-Research Innovation Center Suzhou. Huh7, HepG2 and 293T cell lines were cultured in Dulbecco's modified Eagle medium (DMEM, Gibco). Hep3B and SK-Hep-1 ​cell lines were cultured in Minimum Essential Medium (MEM, Gibco) with sodium pyruvate (Sigma-Aldrich) and non-essential amino acids (Sigma-Aldrich). Li7 cell line was cultured in RPMI 1640 medium (Gibco) with sodium pyruvate. CLC13 cell line was cultured in RPMI 1640 medium with 40 ​ng/mL EGF (PEPROTECH) and ITS (Insulin-Transferrin-Selenium Supplement, Biogems). All medium were supplemented with 10% fetal bovine serum (FBS, ExCell Bio) and 1% penicillin-streptomycin (Solarbio). All cells were authenticated by short tandem repeat (STR) profiling and incubated under 5% CO_2_ at 37 ​°C. Bafilomycin A1(Baf-A1, HY-100558) was purchased from MedChemExpress.

### 2.2 Antibodies, plasmid transfection and generate stable cell lines

Primary antibodies anti-GLDC (12794S), anti-LC3B (3868S), anti-HA-Tag (3724S), anti-Myc-Tag (2278S) and anti-DYKDDDDK-Tag (Flag, 14793S) were purchased from Cell Signaling Technology. The anti-E-Cadherin (20874-1-AP), anti-N-Cadherin (22018-1-AP) and anti-Vimentin (10366-1-AP) were purchased from Proteintech. The anti-PIK3C3/VPS34 (A12483), anti-Beclin1 (A7353), anti-β-Tubulin (AC021) and anti-β-actin (AC026) were purchased from ABclonal.

HA-tagged GLDC was cloned into the PCDH-GFP-puro vector. The short hairpin (shRNA) used for GLDC knockdown was cloned into the pLKO-puro vector. The primers used in this study were listed in Supplementary Table 1. HA-GLDC^K514Q^ plasmid was constructed by GENERAL BIOL. Flag-VPS34, MYC-ATG14 and HA-Beclin1 were purchased from Addgene. HA-tagged GLDC^K73R^, GLDC^K423R^, GLDC^K447R^ and GLDC^K514R^ plasmids were generously provided by Dr. Hongbing Shu from Wuhan University.

The lentivirus plasmids were co-transfected with psPAX2 and pMD2.G into 293T cells using Polyethylenimine (PEI, Polysciences) to produce lentivirus. The lentivirus was collected, purified with PEG8000 (Sigma-Aldrich) and then transduced into target cells using 8 ​μg/mL polybrene (Millipore) to establish GLDC stable silencing and overexpressed HCC cell lines.

### 2.3 Immunohistochemistry (IHC)

The HCC tissue microarray was obtained from Outdo Biotech Company (HLivH150CS05) and IHC assay was performed using primary antibody anti-GLDC (24827-1-AP, Proteintech) at 4 ​°C overnight. The secondary antibody was added and incubated at room temperature for 30 ​min, followed by DAB staining. The stained tissue microarray was scanned and analyzed by Image-Pro Plus software. The integrate optical density (IOD) value was calculated for each tissue, and mean density = (IOD SUM)/area.

### 2.4 Western blotting and Co-immunoprecipitation (co-IP)

Cells were washed with PBS and lysed using RIPA (Beyotime) containing protease inhibitors cocktail (Selleck) and phosphatase inhibitor cocktail (Selleck). Cell lysate was centrifuged at 12000 ​rpm for 10 ​min at 4 ​°C. The supernatant was collected, mixed with loading buffer (Solarbio) and denatured by heating at 98 ​°C for 10 ​min. Sodium dodecyl sulfate-polyacrylamide (SDS-PAGE) gel was used to separate protein and then transferred to PVDF membranes (Cytiva). The membranes were blocked in 5% skim milk (Beyotime) for 2h at room temperature and incubated with primary antibodies overnight at 4 ​°C. After washing in TBST for 4 times, the membranes were incubated with secondary antibodies (7074S/7076S, CST) for 2 h at room temperature. The protein bands were visualized using ECL detection kit (Proteintech).

For Co-IP, cells were washed twice with PBS and lysed using IP lysis buffer (Beyotime) containing protease inhibitors cocktail and phosphatase inhibitor cocktail on ice. 10% of total lysate was used as input group. The remaining lysate was incubated with anti-HA or anti-FLAG magnetic beads (Selleck) overnight at 4 ​°C. The target protein was eluted using loading buffer (Solarbio) after heating at 98 ​°C for 10 ​min.

### 2.5 RNA sequencing

Total RNA was extracted with RNAprep pure cell kit (TIANGEN) from the scramble and shGLDC cells. After RNA quality assessment, the single-stranded cDNA library was constructed and sequencing was performed by BGI Genomics. The sequencing data was analyzed using DESeq2 package to identify differentially expressed genes. The Kyoto Encyclopedia of Genes and Genomes (KEGG) and Gene Set Enrichment Analysis (GSEA) were carried out using Dr. Tom platform from BGI.

### 2.6 Quantitative real-time PCR(qPCR)

Total RNA was isolated with the total RNA extraction kit (Xianfeng Biotechnology) according to the manufacturer's instructions. cDNA was subsequently synthesized through reverse transcription using the ReverTra Ace® RT with gDNA remover kit (TOYOBO). Quantitative real-time PCR was performed using SYBR Green PCR kit (Vazyme). GAPDH was used as control. The primers used for qPCR were listed in Supplementary Table 2.

### 2.7 Cell viability and colony formation assay

In brief, 2000 ​cells per well were seeded in 96-well plate at 37 ​°C and 5% CO_2_. After incubated at indicated time point, the medium was removed and fresh medium containing 10% Cell Counting Kit-8 solution (CCK-8, APExBIO) was added to each well for 3h. Then the absorbance at 450 ​nm and 630 ​nm of each well was determined using a microplate reader (Bio-Tek).

For colony formation assay, 2 ​× ​10^3^ ​cells were seeded in 12-well plate and cultured at 37 ​°C and 5% CO_2_ for about 14 days. The colonies were fixed with 4% paraformaldehyde and stained with 0.1% crystal violet.

### 2.8 Cell scratch assay and transwell assay

The cells were seeded in 6-well plate and cultured until confluent. Scraped a linear wound with sterile tips and removed the cell debris with PBS. The cells were cultured in serum-free medium at indicated time point and photographed at 0h and 48h, respectively.

For transwell assay, 3 ​× ​10^4^ ​cells were resuspended in 500 ​μL serum-free medium in the upper chamber of a Transwell plate (Corning). Then, 500 ​μL medium containing 10% FBS was added to the bottom of the plate. After incubating at 37 ​°C for 24h, cells in the upper chamber were fixed with 4% paraformaldehyde for 30 ​min and stained with 0.1% crystal violet.

### 2.9 Cell senescence assay

β-galactosidase activity was measured using Senescence β-Galactosidase Staining Kit (Beyotime) according to the manufacturer's instructions. The cells were seeded in 12-well plate and cultured at indicated time point. Then cells were fixed and incubated in β-galactosidase staining solution overnight at 37 ​°C under absence of CO_2_ condition. Cells were observed using microscope and photographed for recording.

### 2.10 Animal studies

Four-week-old male BALB/c athymic nude mice were purchased from Vital River Laboratory Animal Technology. All mice were fed under specific pathogen-free conditions at the model animal research center of Shandong University. Mice were distributed into two groups randomly and were injected subcutaneously with Huh7 control and GLDC overexpressed cells (5 ​× ​10^6^ ​cells in 140 ​μL PBS), respectively. Tumor volume was measured every 2 days (volume ​= ​length ​× ​width ​× ​width/2). About 4 weeks after injection, mice were euthanized and tumors were isolated and frozen for the following experiments.

### 2.11 Statistical analysis

Experiments were repeated at least three times independently. Data statistical analysis was performed using GraphPad Prism 8.0. The statistical results were presented as mean ​± ​standard deviation (SD). The data compared between two groups was analyzed using Student's *t*-test. ∗*p* < ​0.05 was considered as statistically significant.

## 3 Results

### 3.1 GLDC inhibits cell proliferation and promotes senescence in HCC

**Fig. 1:**
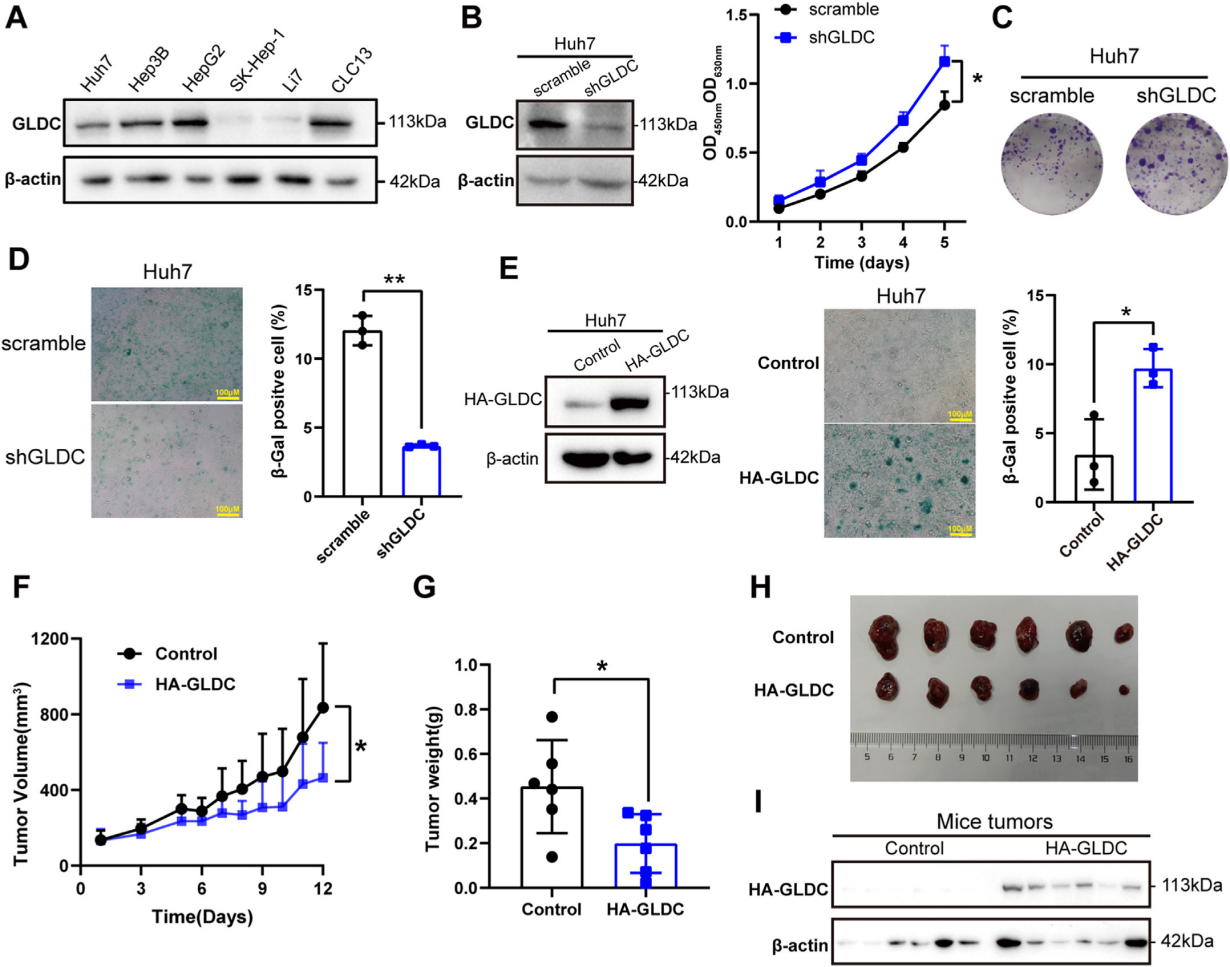
GLDC inhibits cell proliferation and promotes senescence in HCC. (A)The protein expression of GLDC in HCC cell lines. (B) The knockdown efficiency of GLDC and the effect of GLDC depletion on cell proliferation in Huh7 cells. (C) The effect of GLDC depletion on colony formation ability was examined in Huh7 cells. (D) The effect of GLDC knockdown on Huh7 cell senescence. (E) The overexpression efficiency of GLDC and the effect of GLDC on cell senescence were detected in Huh7 cells. (F)Tumor volume curves of control and GLDC overexpressed Huh7 cells after subcutaneous injection to nude mice (n ​= ​6). (G and H) Tumors were weighed and photographed (n ​= ​6). (I) GLDC expression was detected in tumors separated from nude mice. (Compared with control group, ∗*p* ​< ​0.05, compared with scramble group, ∗*p* ​< ​0.05, ∗∗*p* ​< ​0.01. Scale bar: 100 ​μM).

GLDC expression level was determined in several HCC cell lines. We found that GLDC exhibited a relatively high level in Huh7, HepG2, Hep3B and CLC13 ​cells (Fig. 1A). To explore the effect of GLDC on HCC cell viability, we generated stable silencing of GLDC cell lines. And then CCK8 assay was performed in Huh7 and Hep3B cells upon knockdown of GLDC. The results showed that GLDC deprivation significantly increased cell proliferation (Fig. 1B and S1A). The colony formation assay showed that GLDC knockdown increased the number of cell colonies in Huh7 and HepG2 cells (Fig. 1C and S1B). β-galactosidase (β-gal) staining was carried out to determine the effect of GLDC on cell senescence. GLDC knockdown decreased β-gal-positive cells, while GLDC overexpression increased β-gal-positive cells in Huh7 cells (Fig. 1D and E), suggesting that GLDC promotes cell senescence in HCC. We further used xenograft model to detect the role of GLDC on tumor growth. Control and GLDC overexpressed Huh7 cells were subcutaneously injected into BALB/c nude mice, respectively. As expected, GLDC overexpression sharply inhibited tumor volume and diminished tumor weight (Fig. 1F–H). In addition, Western blotting was performed to confirm GLDC expression in mice tumors and GLDC protein was overexpressed compared with control group (Fig. 1I). Taken together, GLDC inhibits cell proliferation, reduces tumor growth and induces cell senescence in HCC.

### 3.2 GLDC inhibits cell migration and EMT *in vitro*

**Fig. 2:**
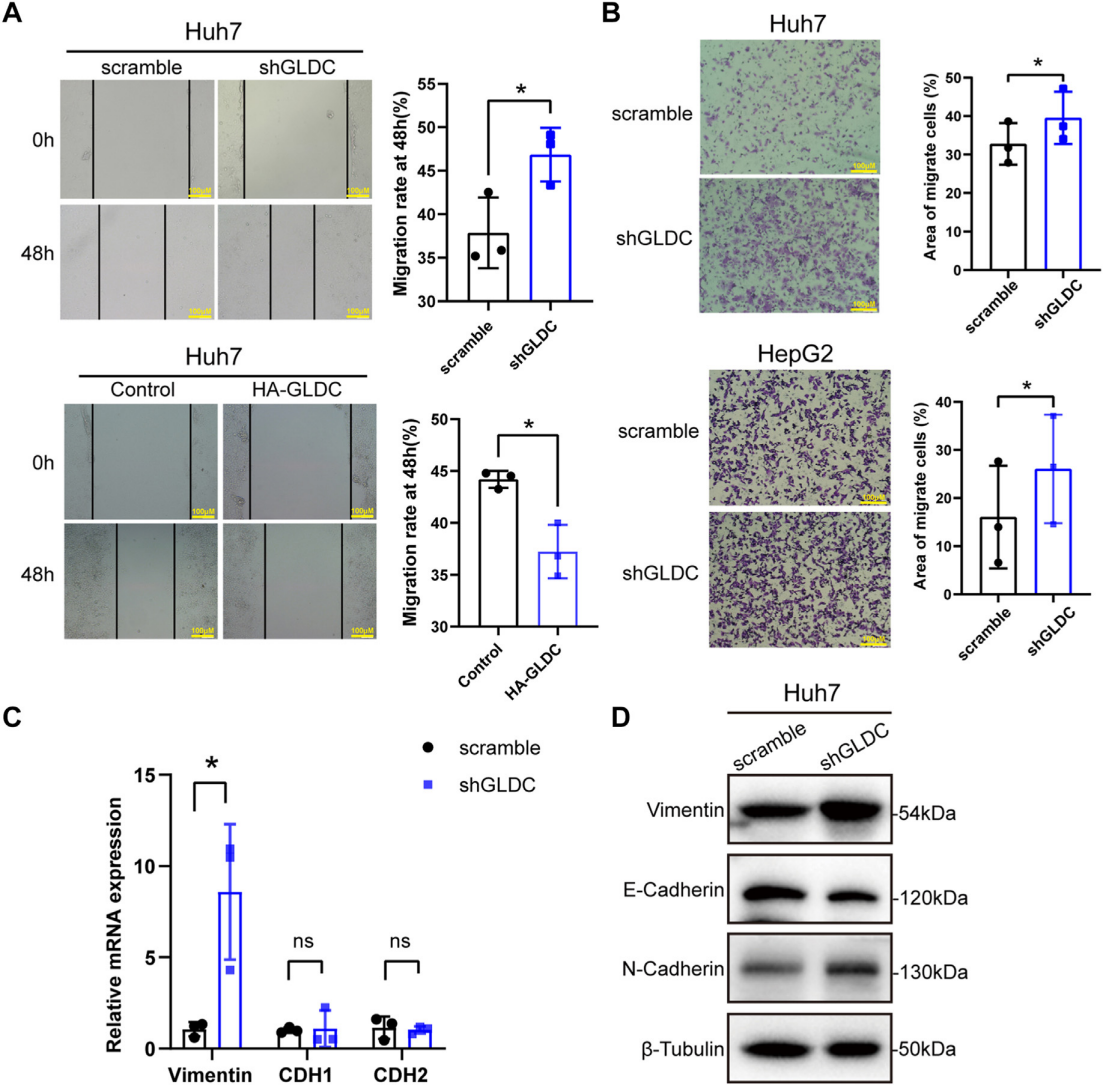
GLDC inhibits HCC cell migration and EMT *in vitro*. (A) The effect of GLDC knockdown and overexpression on migration in Huh7 cells. (B) The impact of GLDC knockdown on cell migration in Huh7 and HepG2 cells. (compared with scramble group, paired Student's *t*-test, ∗*p* ​< ​0.05) (C and D) The effect of GLDC knockdown on mRNA and protein level of EMT markers Vimentin, N-Cadherin, and E-Cadherin in Huh7 cells, respectively. (Compared with control group, ∗*p* ​< ​0.05, compared with scramble group, ∗*p* ​< ​0.05. Scale bar: 100 ​μM).

To explore the effect of GLDC on cell migration, we performed wound-healing assay *in vitro*. The results showed that GLDC knockdown significantly promoted cell migration, while GLDC overexpression inhibited cell migration compared with the control group (Fig. 2A). In addition, transwell assay showed that GLDC depletion enhanced the number of migrating cells in Huh7 and HepG2, indicating that GLDC displays a noticeable inhibitory effect on HCC cell migration (Fig. 2B). EMT program plays a vital role in tumor metastasis [28,29]. Then, we examined the mRNA and protein levels of EMT-related markers, including Vimentin, E-Cadherin and N-Cadherin. The results showed that GLDC knockdown upregulated mRNA level of Vimentin and protein levels of Vimentin and N-Cadherin, both of which were mesenchymal cell markers. In addition, GLDC depletion downregulated protein expression of the epithelial cell marker E-Cadherin, suggesting that GLDC suppresses cell migration and EMT program in HCC (Fig. 2C and D).

### 3.3 GLDC interacts with VPS34 and promotes autophagy

**Fig. 3:**
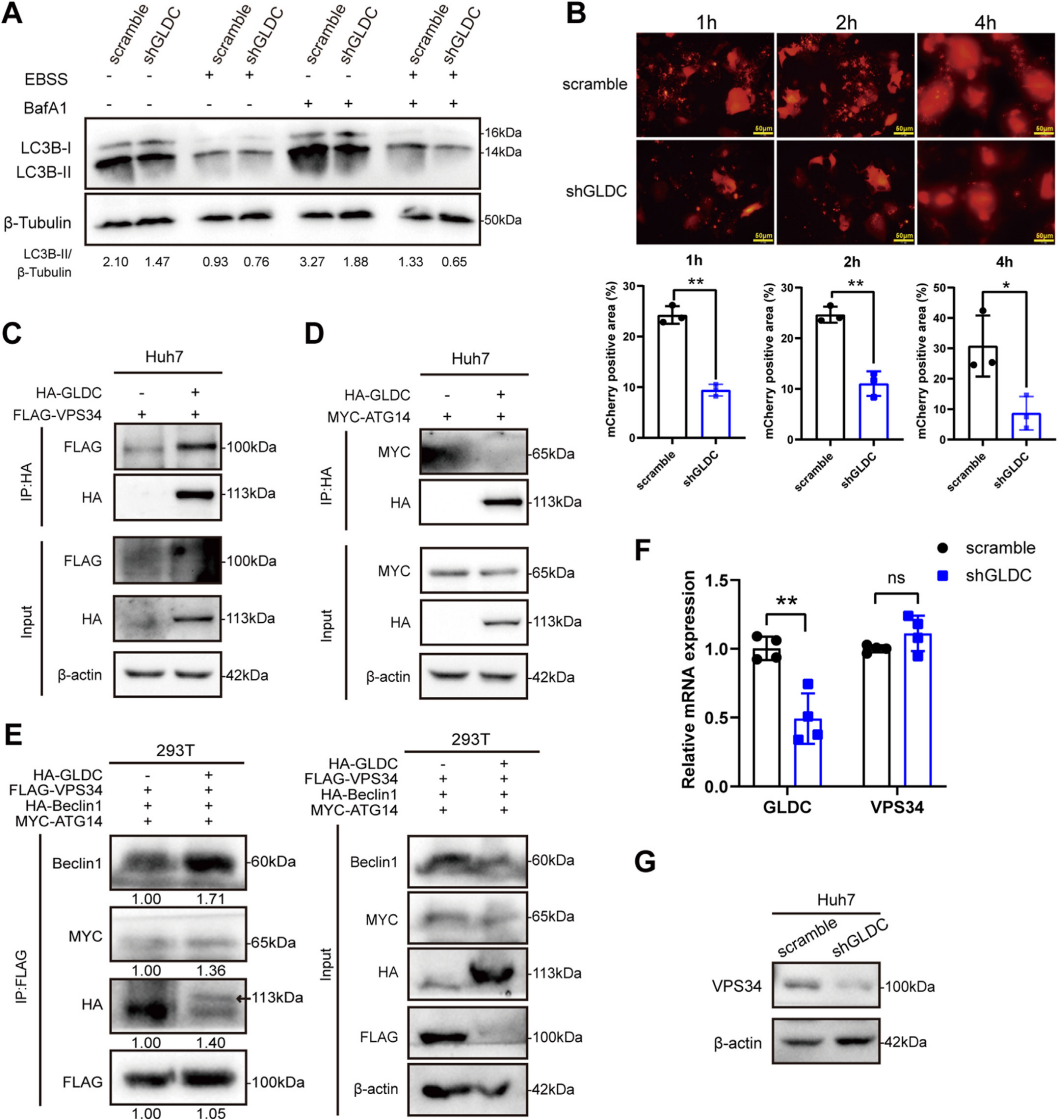
GLDC interacts with VPS34 and promotes autophagy in HCC. (A) The effect of GLDC knockdown on LC3B protein in Huh7 cells upon EBSS starvation and autophagy inhibitor BafA1 conditions. (B) Representative images and statistics analysis of autophagy flux in BafA1-treated GLDC knockdown and scramble Huh7 cells. (C and D) GLDC overexpressed Huh7 cells were transfected with FLAG-VPS34 or MYC-ATG14 plasmid to detect the interaction of GLDC with VPS34 or ATG14. (E) 293T cells were transfected with HA-tagged wild-type GLDC, FLAG-tagged VPS34, HA-tagged Beclin 1 and MYC-tagged ATG14, to detect the effect of GLDC on the interaction of VPS34 with Beclin 1 or ATG14. (F and G) The expression level of VPS34 in GLDC knockdown Huh7 cells. (Compared with scramble group, ∗*p* ​< ​0.05, ∗∗*p* ​< ​0.01. Scale bar: 50 ​μM).

Autophagy modulates several tumor-related signaling pathways and inhibits cell malignant features, such as cell proliferation, EMT and metastasis [22,30]. It has been reported that miRNA-30 targets GLDC and is involved in autophagy modulation in HCC [19]. However, the underlying mechanism by which GLDC induces autophagy remains unclear. We determined the effect of GLDC on autophagy in Huh7 cells. The results showed that the ratio of LC3B-II to β-tubulin in GLDC knockdown group decreased compared with that in scramble cells upon Earle's Balanced Salt Solution (EBSS) starvation and treated with autophagy inhibitor Bafilomycin A1 (Baf-A1) (Fig. 3A). We then transfected mCherry-LC3B plasmid into EBSS-treated Huh7 cells, and found that GLDC knockdown reduced the formation of LC3B red fluorescence (Fig. 3B). Then, we elucidate the underlying mechanism by which GLDC modulates autophagy in HCC cells. We detected whether GLDC may interact with ATG14 or VPS34, which play crucial roles in autophagy initiation. The co-IP experiments found that VPS34, rather than ATG14, interacted with GLDC in Huh7 cells (Fig. 3C and D). It has been reported that VPS34 forms an autophagy complex with Beclin1 and ATG14 to promote the progression of autophagy at the initiation stage [25,31]. We further examined whether GLDC affects the interaction of VPS34 with Beclin1 and ATG14 in 293T cells. Importantly, the results showed that GLDC overexpression enhanced the interaction of VPS34 with Beclin1 and ATG14 in 293T cells (Fig. 3E). Furthermore, GLDC silencing decreased the protein level of VPS34 in Huh7 cells, whereas GLDC didn't affect the mRNA level of VPS34 (Fig. 3F and G).

### 3.4 GLDC K514 acetylation enhances the interaction of GLDC-VPS34

**Fig. 4:**
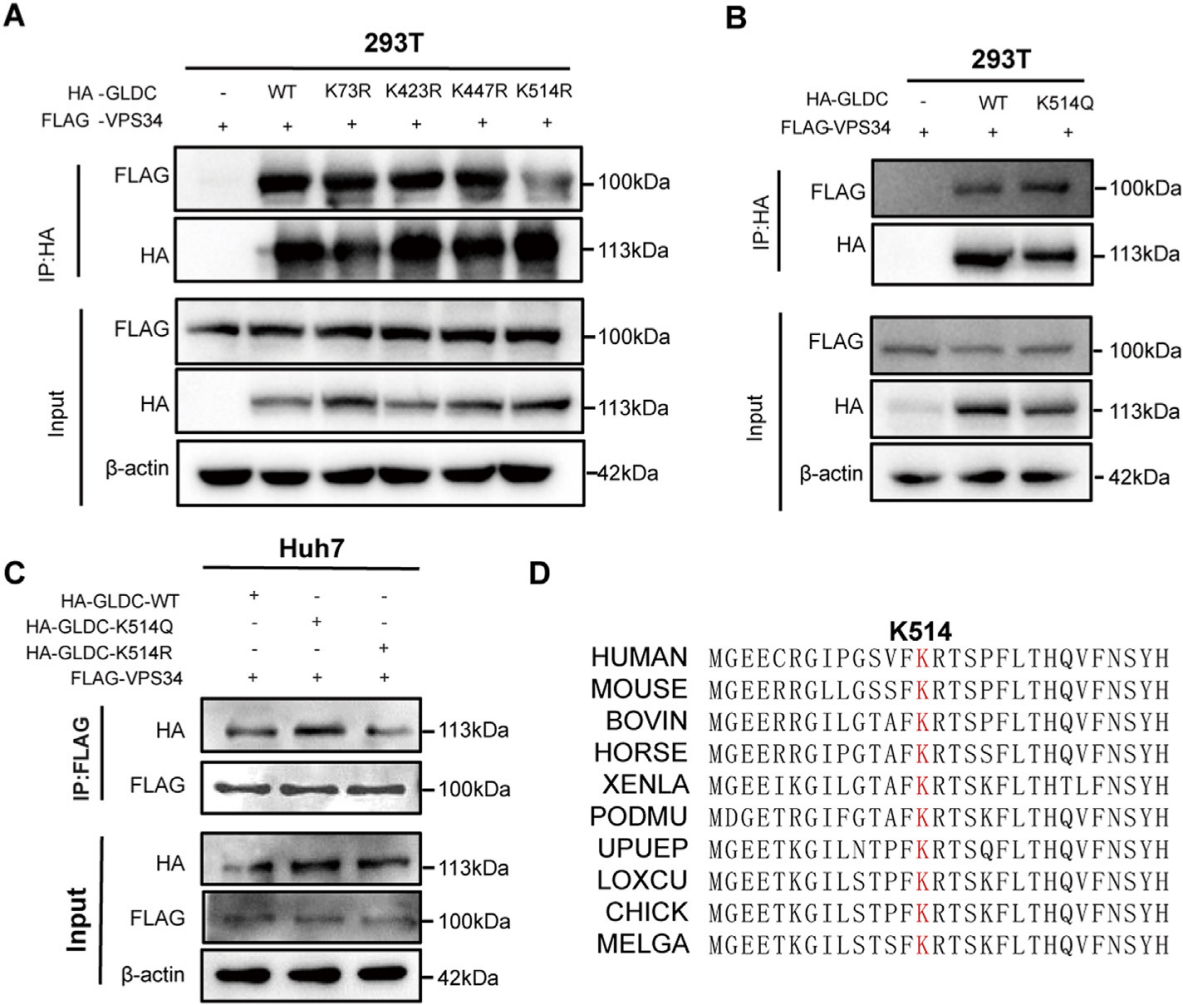
GLDC acetylation at K514 promotes the interaction of GLDC-VPS34. (A) The effect of GLDC K73R, K423R, K447R and K514R mutants on the interaction between GLDC and VPS34 in 293T cells. (B) 293T cells were transfected with HA-tagged wild-type GLDC or GLDC^K514Q^ plasmid to detect the effect of GLDC K514 acetylation on the interaction of GLDC-VPS34. (C) Huh7 cells were transfected with HA-tagged wild-type GLDC, GLDC^K514R^ or GLDC^K514Q^ plasmid to detect the effect of GLDC K514 acetylation on the interaction between GLDC and VPS34. (D) Sequence alignment of GLDC in 10 different species.

One previous study has revealed that GLDC acetylation participates in pyrimidines synthesis and glioma tumorigenesis [15]. We predicted potential acetylation sites of GLDC protein using an online phosphosite-plus website (Fig. S2). To further explore the role of GLDC acetylation in HCC, four mutants (GLDC^K73R^, GLDC^K423R^, GLDC^K447R^ and GLDC^K514R^) were constructed to mimic GLDC lysine deacetylation. We transfected HA-tagged wild-type GLDC plasmid and above-mentioned acetylation-disabled mutants into 293T cells and determined whether GLDC acetylation affects its binding to VPS34. The data showed that only GLDC acetylation mutants at K514 attenuated the interaction of GLDC and VPS34, while the association of GLDC K73R, K423R or K447R mutant and VPS34 was not affected (Fig. 4A). GLDC^K514Q^ mutant were created to mimic GLDC lysine acetylation, and the effect of GLDC acetylation on its interaction with VPS34 was detected. Intriguingly, GLDC K514 acetylation promotes the interaction between GLDC and VPS34 (Fig. 4B). Similarly, we observed that GLDC^K514Q^ enhanced GLDC-VPS34 interaction, while GLDC^K514R^ abolished their interaction in Huh7 cells (Fig. 4C), indicating that GLDC acetylation at K514 is partly responsible for enhanced binding to VPS34. Meanwhile, the GLDC K514 is conserved in different species (Fig. 4D).

### 3.5 GLDC modulates cell cycle, lipid metabolism and ferroptosis pathways

**Fig. 5:**
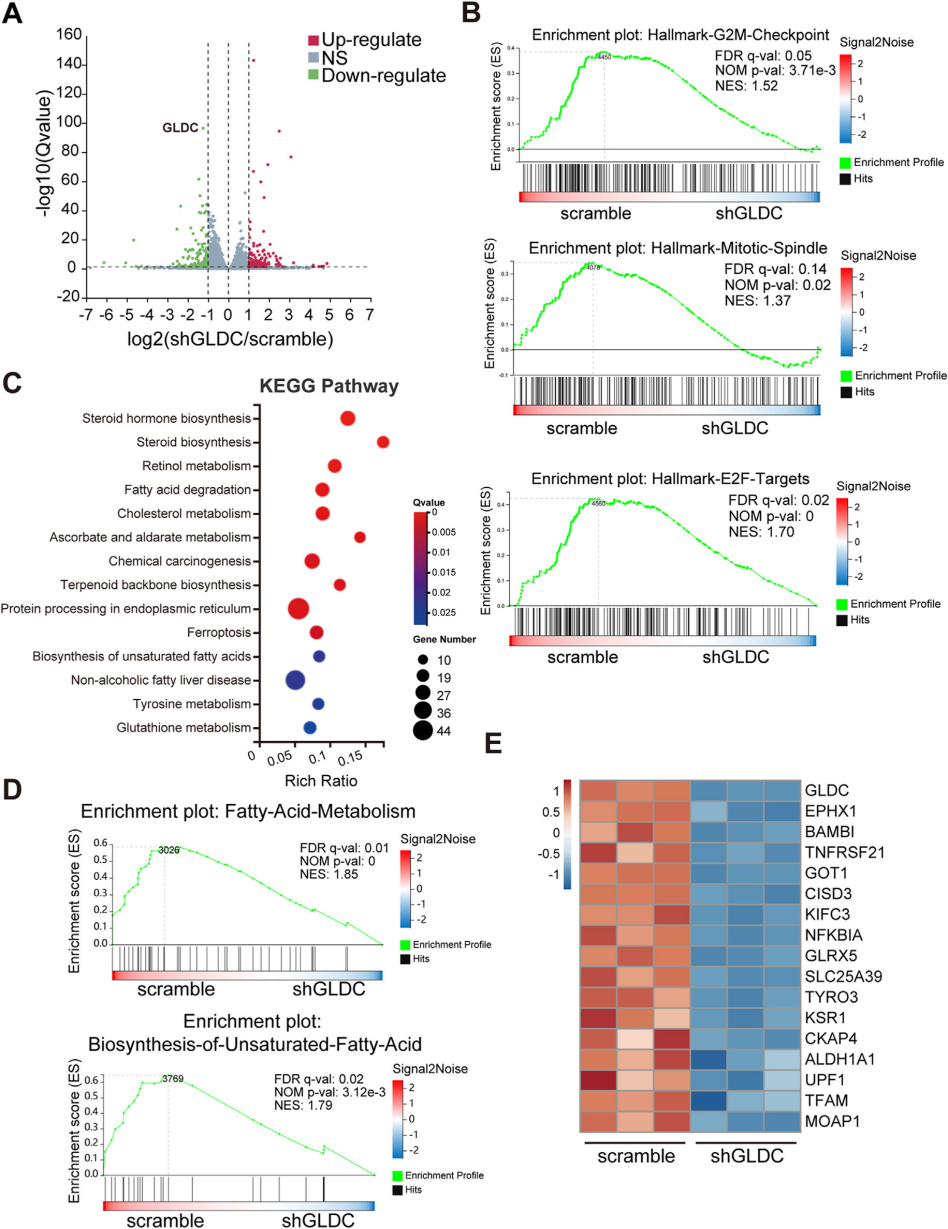
GLDC modulates cell cycle, lipid metabolism and ferroptosis pathways. (A) Volcano plot was presented using the altered genes in GLDC knockdown compared with scramble cells detected by RNA sequencing. (B) GSEA analysis showed enriched pathways in G2M checkpoint, mitotic spindle and E2F targets. (C) KEGG enrichment pathway analysis of altered genes in lipid metabolism-related pathways and ferroptosis pathways. (D) GSEA analysis showed enriched pathways in fatty acid metabolism and biosynthesis of unsaturated fatty acid. (E) Heatmap showed the top significantly differentially expressed genes associated with lipid metabolism and cell cycle progression.

To dissect the downstream target genes modulated by GLDC, we performed RNA sequencing analysis in GLDC knockdown cells. Transcriptomic analysis showed that the expression levels of 122 genes were significantly upregulated and 115 genes were downregulated (Fig. 5A). Gene Set Enrichment Analysis (GSEA) indicated that cell cycle-related gene signature was positively enriched in GLDC knockdown group, including E2F targets, mitotic spindle and G2M checkpoint (Fig. 5B), which partially accounts for the suppression of cellular proliferation mediated by GLDC. The Kyoto Encyclopedia of Genes and Genomes (KEGG) enrichment pathway analysis showed that GLDC was closely associated with lipid metabolism and ferroptosis (Fig. 5C and D). Sixteen differentially expressed genes were presented in the heat map, which were involved in lipid metabolism and cell cycle progression (Fig. 5E).

### 3.6 GLDC is lowly expressed and predicts poor prognosis in HCC

**Fig. 6:**
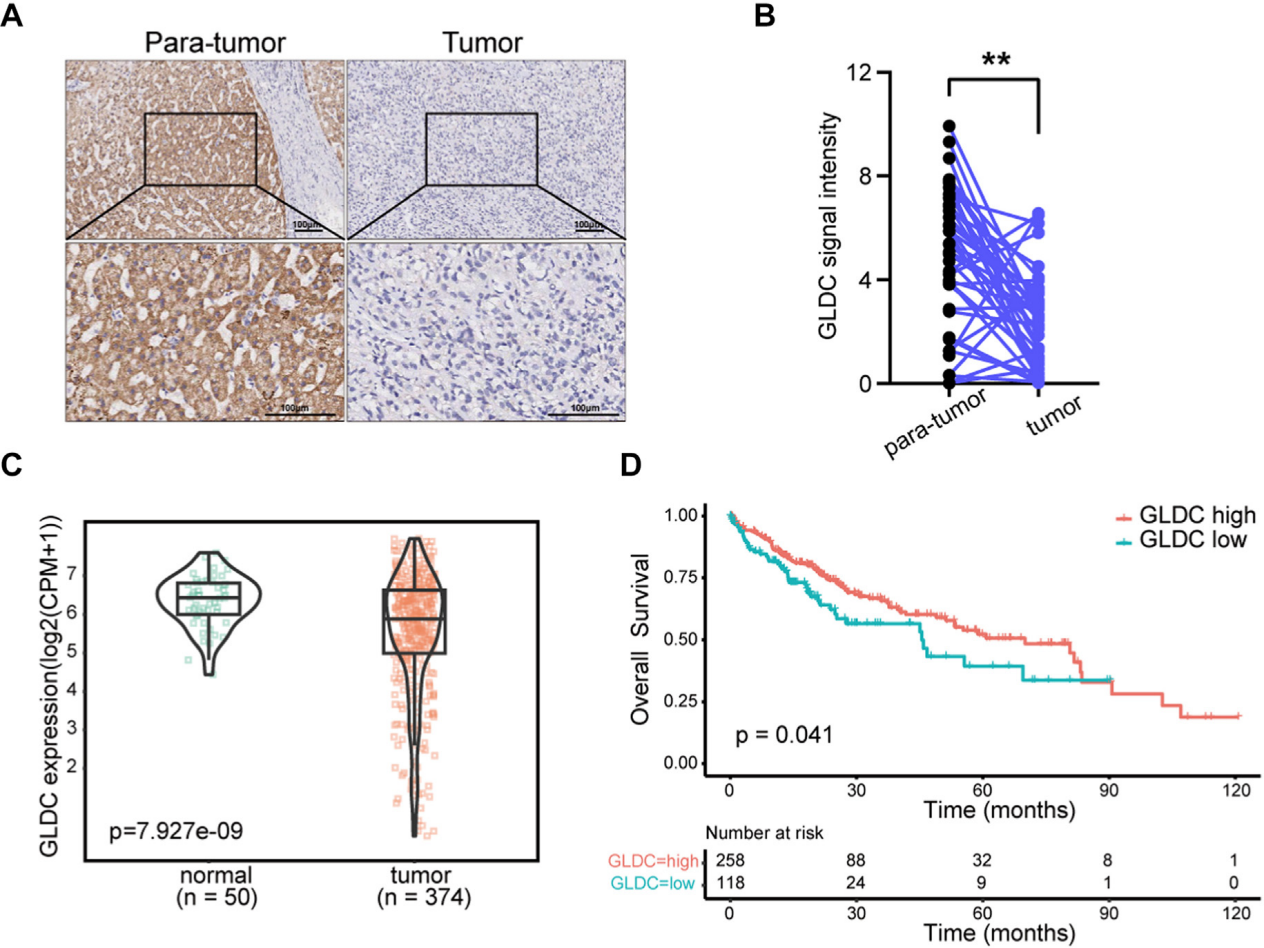
GLDC is lowly expressed and predicts poor prognosis in HCC. (A and B) Representative images of IHC staining and stain score of GLDC in HCC cancer tissues and para-tumor tissues. (C) The mRNA expression level of GLDC in HCC patients and normal people by analysis of TCGA data. (D). The overall survival rate of HCC patients with different expression levels of GLDC. (Compared with para-tumor group, paired Student's *t*-test, ∗∗*p* ​< ​0.01. Scale bar: 100 ​μM).

To further explore the role of GLDC in HCC, we performed immunohistochemistry staining using tissue microarray and detected GLDC protein expression in HCC and para-tumor tissue samples. We found that GLDC expression was significantly downregulated in tumor tissues (Fig. 6A and B). We then analyzed the mRNA level of GLDC using TCGA database. As expected, the expression level of GLDC was significantly decreased in tumors compared to non-tumor liver tissues (Fig. 6C). Moreover, Kaplan-Meier survival analysis indicated that the overall survival rate of HCC patients with low GLDC expression was significantly reduced (Fig. 6D). These data demonstrated that the decreased expression of GLDC is positively correlated with poor prognosis in HCC.

## 4 Discussion

Metabolic reprogramming is a common feature of cancer cells, as well as a critical characteristic, which differentiates them from normal cells [32]. The alteration in metabolic patterns enables cells to survival under external pressures [33]. Glycine metabolism provides one carbon unit and nucleotides, which is closely related to the occurrence and development of tumors. GLDC is a key rate-limiting enzyme in glycine metabolism and functions as an oncogene or tumor suppressor in different cancer types [34]. We aimed to investigate the effect of GLDC on cell proliferation, migration, tumor growth and autophagy and clarified the molecular mechanism of GLDC regulating autophagy in HCC.

In this study, we found that knockdown of GLDC promoted the cell viability, colony formation and tumor growth. Upon depletion of GLDC, glycine may accumulate in HCC cells, which is partly converted to serine for supporting HCC cell proliferation and energy supply. To elucidate the molecular mechanism of GLDC in cell proliferation, high-throughput RNA sequencing and GSEA analysis showed that the differentially expressed genes regulated by GLDC were mainly enriched in mitotic process, cell cycle checkpoint and fatty acid metabolism pathways. In addition, SA-β-gal staining showed that GLDC promoted HCC cell senescence, suggesting that cells may present a state of impaired proliferation and cell cycle arrest by GLDC. We detected the migration ability of HCC cells by wound healing assay and transwell assay and found that GLDC knockdown promoted the migration ability of HCC cells. Then, EMT-related marker molecules were examined, and GLDC significantly downregulated Vimentin levels, suggesting that GLDC inhibition of HCC cell migration may be related to its regulation of Vimentin. However, how GLDC regulates Vimentin expression deserves further investigation. Consistent with our finding, GLDC inhibits HCC migration by regulating the stability of cytoskeletal protein cofilin [18].

Autophagy plays a dual role in cancer development [26,35]. On the one hand, it facilitates tumor cell to survive in adverse environments, on the other hand, during the early stages of tumor development, autophagy can suppress tumorigenesis by modulating pro-tumor factors [36,37]. The role of GLDC in HCC is complex and sometimes uncertain. On the one hand, GLDC inhibits biological processes such as migration and autophagy in HCC. Specifically, GLDC downregulation enhances cell migration and intrahepatic metastasis by ROS-mediated ubiquitination of cofilin [18]. Besides, GLDC induces cell autophagy through epigenetic silencing by miR-30d-5p [19]. On the other hand, GLDC promotes mitochondrial protein lipoylation and tumor growth, indicating an oncogene role [38]. In our study, we found that GLDC acted as a tumor suppressor and revealed the downstream mechanism by which induced autophagy in HCC. We confirmed that knockdown of GLDC resulted in reduced levels of LC3B-II and significantly decreased autophagic flux compared to control cells. More importantly, we first revealed the underlying mechanism of autophagy modulation by GLDC in HCC. Through co-immunoprecipitation assay, we identified the interaction between GLDC and one of key autophagy initiation protein VPS34. Furthermore, knockdown of GLDC significantly downregulated the expression of VPS34. Previous studies have demonstrated that VPS34 expression is down-regulated in HCC cells, and modulates epithelial-mesenchymal transition and cell migration [39,40], suggesting that VPS34 may serve as a downstream gene of GLDC and mediate its inhibitory effect on HCC cell migration. As an upstream component of autophagy, the function of VPS34 relies not only on phosphorylation but also on cooperative interactions with complex chaperones such as Beclin1, ATG14, and UVRAG to facilitate autophagosome formation and maturation [24,41,42]. Therefore, we hypothesized that GLDC might influence autophagy progression by modulating the interaction among proteins within the VPS34 complex. We found that GLDC indeed modulate the progression of autophagy by regulating the VPS34 complex protein-protein interaction. The protein acetylation plays a vital role in protein interaction. Interestingly, we demonstrated that GLDC K514 acetylation enhanced its interaction with VPS34 to induce autophagy and GLDC^K514R^ reduced its interaction.

The low GLDC expression is associated with poor prognosis in HCC, thus it may be as a prognostic biomarker for HCC. GLDC acetylation enhanced its interaction with VPS34, thereby promoting autophagy and inhibiting HCC progression. Thus, developing autophagy inducers and compounds of enhancing interaction of GLDC-VPS34 may represent a potential therapeutic strategy for HCC.

## 5 Limitations

In this study, GLDC acetylation at K514 affected its interaction with VPS34. Thus, GLDC acetylation level at K514 need to be determined in clinical HCC samples. RNA sequencing results revealed that GLDC was closely associated with the cell cycle, lipid metabolism, and ferroptosis. How GLDC regulates above-mentioned biological processes in HCC requires further investigation. This will provide a more comprehensive understanding of the role of GLDC in HCC progression and its potential as a therapeutic target.

## 6 Conclusion

In summary, our finding provides critical insights into the mechanism of GLDC in HCC aggression and autophagy. We reveal a positive association between decreased GLDC expression and a poor prognosis in HCC patients. GLDC acetylation at K514 enhances its interaction with VPS34, thereby tremendously inhibiting malignant features such as cell proliferation, migration, autophagy and tumor growth in HCC.

## CRediT authorship contribution statement

**Zan Song:** Writing – original draft, Visualization, Validation, Methodology, Investigation, Formal analysis. **Hao Dong:** Visualization, Validation, Methodology, Investigation, Formal analysis. **Kailing Zhang:** Visualization, Methodology, Investigation. **Bingke Qiao:** Visualization, Methodology, Investigation. **Leilei Li:** Visualization, Investigation. **Zhicheng Zhang:** Visualization, Investigation. **Zhili Fan:** Visualization, Investigation. **Jing Li:** Visualization, Investigation. **Yu Li:** Visualization, Investigation. **Mengfei Liu:** Visualization, Investigation. **Ying Liu:** Visualization, Investigation. **Xinyu Gu:** Visualization, Investigation. **Tao Zhang:** Writing – review & editing, Supervision, Resources, Project administration, Methodology, Funding acquisition, Conceptualization.

## Ethical approval

All animal experiments were approved by the Animal Care and Use Committee of Shandong University. (Approval No. 230067).

## Data availability

Data will be made available on request.

## Declaration of generative AI in scientific writing

Not applicable.

## Funding information

This work was supported by the National Natural Science Foundation of China (82104218), the Taishan Scholars Program (TSQN201909033) of Shandong Province and the program for Multidisciplinary Research and Innovation Team of Young Scholars of Shandong University (2020QNQT002).

## Declaration of competing interest

The authors declare that they have no known competing financial interests or personal relationships that could have appeared to influence the work reported in this paper.
