# Corrigendum to “GLDC interacts with VPS34 to inhibit tumorigenesis and epithelial-mesenchymal transition in hepatocellular carcinoma” [Adv. Pharmaceut. Sci. (3), (2025), 100072]

**DOI:** 10.1016/j.pscia.2025.100081

**Published:** 2025-09-07

**Authors:** Zan Song, Hao Dong, Kailing Zhang, Bingke Qiao, Leilei Li, Zhicheng Zhang, Zhili Fan, Jing Li, Yu Li, Mengfei Liu, Ying Liu, Xinyu Gu, Tao Zhang

**Affiliations:** a Institute of Immunopharmaceutical Sciences, State Key Laboratory of Discovery and Utilization of Functional Components in Traditional Chinese Medicine, NMPA Key Laboratory for Technology Research and Evaluation of Drug Products, School of Pharmaceutical Sciences, Cheeloo College of Medicine, Shandong University, Jinan, Shandong, China; b Department of Pharmacy, Tengzhou Central People's Hospital, Shandong, China

The authors regret:

The “autophagy” in the last sentence of Conclusion is redundant. Therefore, this section should be revised as below.

Conclusion.

In summary, our finding provides critical insights into the mechanism of GLDC in HCC aggression and autophagy. We reveal a positive association between decreased GLDC expression and a poor prognosis in HCC patients. GLDC acetylation at K514 enhances its interaction with VPS34, thereby tremendously inhibiting malignant features such as cell proliferation, migration and tumor growth in HCC.

The authors would like to apologise for any inconvenience caused.

