# Supplementary material for: GLDC interacts with VPS34 to inhibit tumorigenesis and epithelial-mesenchymal transition in hepatocellular carcinoma
Source: Pharm Sci Adv. 2025 Apr 25;3:100072. doi: 10.1016/j.pscia.2025.100072 (PMC12709974; doi:10.1016/j.pscia.2025.100072)
Supplement: Multimedia component 1 [file mmc1.docx]

## Supplementary materials

**Table 1** **The sequences used in this study.**

| Name | Sequence（5’-3’） |
| --- | --- |
| PLKO.1-shGLDC1 | F:CCGGTGAATCTCTGTCAAGGTAAACTCGAGTTTACCTTGACAGAGATTACATTTTG  R:AATTCAAAAATGTAATCTCTGTCAAGGTAAACTCGAGTTTACCTTGACAGAGATTACA |
| PLKO.1-shGLDC2 | F:CCGGGAAGTTTATGAGTCTCCATTTCTCGAGAAATGGAGACTCATAAACTTCTTTTTG  R:AATTCAAAAAGAAGTTTATGAGTCTCCATTTCTCGAGAAATGGAGACTCATAAACTTC |
| PLKO.1-scramble | F:CCGGCCTAAGGTTAAGTCGCCCTCGCTCGAGCGAGGGCGACTTAACCTTAGGTTTTTG  R:AATTCAAAAACCTAAGGTTAAGTCGCCCTCGCTCGAGCGAGGGCGACTTAACCTTAGG |
| HA-GLDC | F:CGCTCTAGACACCATGTACCCATACGATGTTCCAGATTACGCTCAG  R: CAAGCTAGCCTAAGAAGACGCCCTCTTTTGTTCAG |

**Table 2 The primer sequences used in this study.**

| Gene | Species | Sequence（5’-3’） |
| --- | --- | --- |
| *GLDC* | Human | F: ACGTGTGTGACCTCATCCATCAACA  R: GTGGCTTGTTTAAGACCCTTGCCTC |
| *CDH1* | Human | F: GCCTCCTGAAAAGAGAGTGGAAG  R: TGGCAGTGTCTCTCCAAATCCG |
| *CDH2* | Human | F: CCTCCAGAGTTTACTGCCATGAC  R: GTAGGATCTCCGCCACTGATTC |
| *Vimentin* | Human | F: AGGCAAAGCAGGAGTCCACTGA  R: ATCTGGCGTTCCAGGGACTCAT |
| *PIK3C3* | *Human* | *F: GCGTTCTTTGCTGGCTGCACAA*  *R: CTCCAAGCAATGCCTGTAGTCTC* |
| *GAPDH* | *Human* | *F: GGAGCGAGATCCCTCCAAAAT*  *R: GGCTGTTGTCATACTTCTCATGG* |


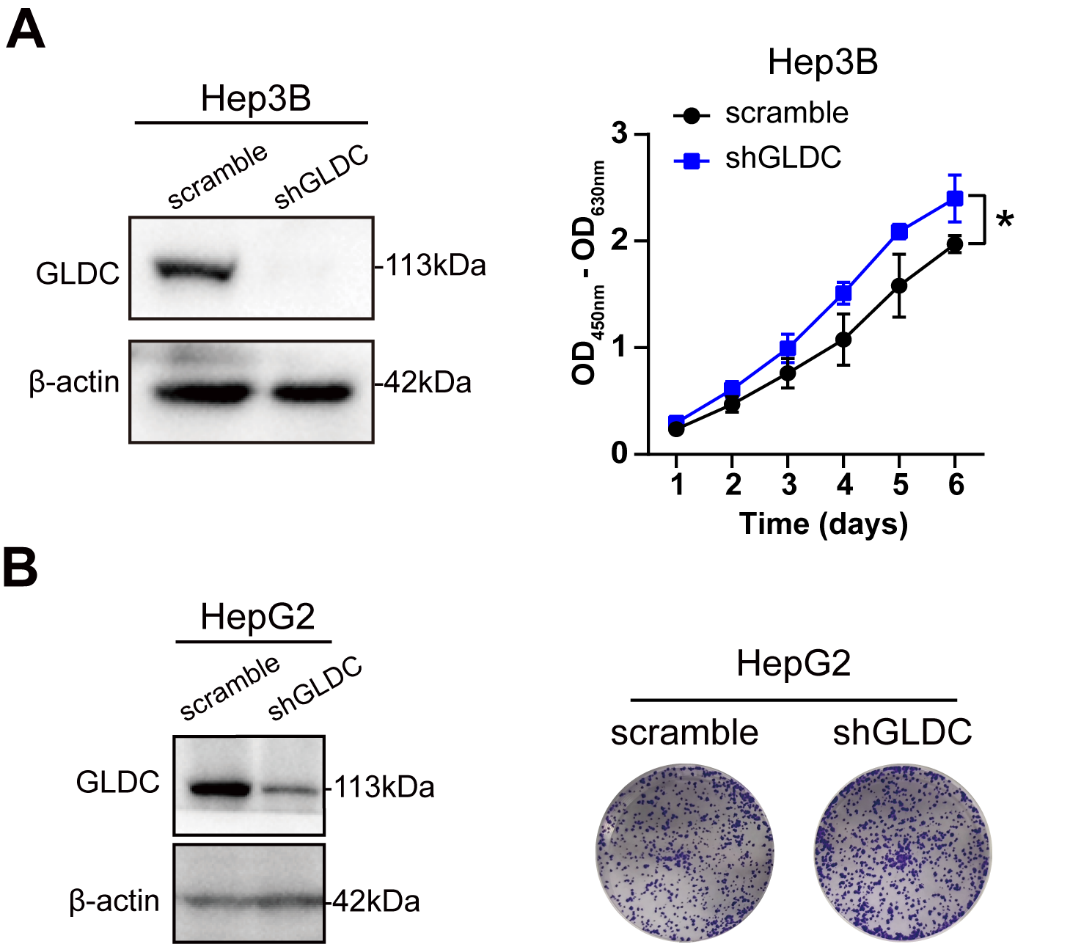


Fig S1. GLDC inhibits the proliferation of HCC cells. (A) Construction of GLDC knockdown Hep3B cell line to detect the effect of GLDC knockdown on cell proliferation. (B) Construction of GLDC knockdown HepG2 cell line and detect the impact of GLDC knockdown on cell colony formation ability. (Compared with the scramble group, *p<0.05)


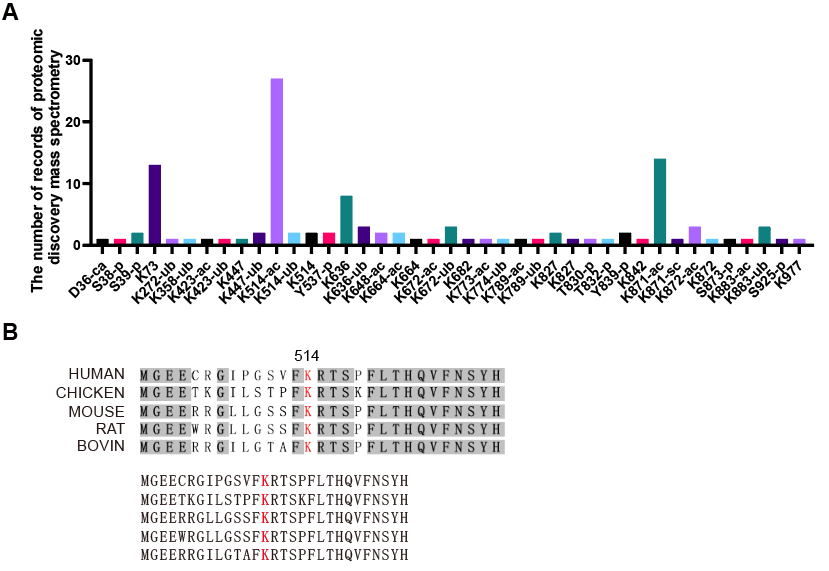
Fig S2. GLDC deacetylation promotes the interaction of GLDC-VPS34. (A) Acetylation sites on GLDC (‘https://www.phosphosite.org/proteinAction.action?id=15033’).
